# Decreased Expression of Inhibitor of Caspase-Activated DNase (ICAD) in Renal Cell Carcinoma – Tissue Microarray of Human Samples

**DOI:** 10.15586/jkcvhl.2016.47

**Published:** 2016-03-22

**Authors:** Retnagowri Rajandram, Azad H. A. Razack, Keng Lim Ng, Glenda C. Gobe

**Affiliations:** 1 Department of Surgery, Faculty of Medicine, University Malaya, Kuala Lumpur, Malaysia; 2 University Malaya Cancer Research Institute (UMCRI), and 3 University Malaya Medical Centre, Kuala Lumpur, Malaysia; 4 Centre for Kidney Disease Research, School of Medicine, The University of Queensland, Translational Research Institute, Brisbane, Australia.

**Keywords:** apoptosis, ICAD, kidney cancer, renal cell carcinoma, tissue microarray

## Abstract

Although primary localised tumours of renal cell carcinoma (RCC) can be treated relatively successfully with surgery, metastatic RCC has poor prognosis because of late diagnosis and resistance to therapies. In the present study, we were interested in profiling the protein expression of “inhibitor of caspase-activated DNase” (ICAD), an apoptosis inhibitor, in kidney cancer and its paired normal kidney. Immunohistochemistry with automated batch staining and morphometry using digital pathology were used to compare ICAD in 121 RCC specimens with their paired normal kidney tissue. Tissue microarray of formalin-fixed, paraffin-embedded archival tissue was used. Intensity and localisation of ICAD were compared between normal and cancer samples, and against grading within the cancers. The results demonstrated that, in this cohort, ICAD was highly expressed in the proximal tubular epithelium of normal kidney, and significantly decreased in clear cell RCC tissue (p < 0.05) as well as other subtypes of RCC (p < 0.01) compared with normal kidney. There was a tendency towards nuclear localisation of ICAD in clear cell RCC, but not in other subtypes of RCC. No significant association was found between ICAD intensity and grade of RCC. In summary, down-regulation of ICAD occurs in RCC. ICAD normally inhibits DNA fragmentation and apoptosis; thus, its down-regulation was unexpected in a cancer known for its resistance to apoptosis. However, these RCC samples were from primary, not metastatic, RCC sites, and down-regulated ICAD may be part of a progressive pathway that promotes RCC metastasis.

## Introduction

Renal neoplasms account for approximately 3% of the total human cancers. Renal cell carcinomas (RCCs), the major proportion of renal neoplasms, are the tenth leading cause of cancer mortality in Western industrialised countries, including Australia (1–3). RCC represents on average 90% of the renal neoplasms in adults of both sexes worldwide (3). Although surgery is a successful curative treatment for localised RCC, metastasis is common and these metastases are resistant to cancer therapies. Improving therapy-associated kidney cancer regression would be of great benefit to patients. Increased knowledge of the underlying molecular characteristics of RCC will result in the identification of molecular pathways involved in tumour growth and metastasis, and resistance to therapies, and expedite development of targeted therapies, and may also identify useful markers of RCC development and progression.

The failure in initiation of apoptosis is a hallmark of treatment-resistant tumours (4, 5). However, understanding the intricacies of how cells fail in the apoptotic process is complex and very tissue specific. In most instances, induction of apoptosis requires DNA fragmentation. The enzyme responsible for apoptotic DNA fragmentation is caspase-activated DNase (CAD). CAD is normally inhibited by the inhibitor of CAD (ICAD). During apoptosis, the effector caspase, caspase-3, cleaves ICAD, causing CAD to become activated and degradation of nuclear DNA into nucleosomal units to occur (6–8).

CAD and ICAD are members of the cell death–inducing DFF-45 effector (CIDE) protein family (9). Members CIDE-A and CIDE-B promote cell death and DNA fragmentation. They are inhibited by ICAD and activated by CAD. The CIDEs, ICAD and CAD have not yet been widely studied in cancer in general, let alone in RCC. CIDE-B has shown some prospect as a biomarker for cancers. For example, CIDE-B was found to be up-regulated in a proteomic study of cervical cancer after treatment with 5-fluorouracil, and it was also found to interact with chronic hepatitis C virus infection to induce liver cancer (10). Hara et al. (11) found, using a cell culture model of RCC, that when ICAD was over-expressed in tumour cells, it rendered them highly-resistant to therapy-induced apoptosis, an outcome that could possibly result in enhanced tumour progression in an *in vivo* situation (11). In a more recent cell culture study, multiple RCCs were treated with chemotherapy and/or radiotherapy, and RNA microarray technology was used to analyse expression of apoptosis-related genes in resistance to cancer therapy (12). In this investigation, one of the novel genes, not previously characterised in RCC apoptosis, but showing a multi-fold increase in expression in RNA microarray, was ICAD. ICAD could contribute to increased inhibition of caspase-dependent cell death in RCC when they are treated with chemotherapeutics. However, it is also known that cells that lack ICAD, or express a caspase-resistant mutant of ICAD, do not show DNA fragmentation during apoptosis, but may die via other cell death pathways (13, 14).

There have been no published studies of ICAD expression in human patient kidney cancer samples. The aim of this paper was to use a set of archival tissue blocks of RCC, characterised and diagnosed by the WHO classification system of 2004 (15) plus their paired normal tissue, to prepare tissue microarrays (TMAs) and to evaluate and profile ICAD using immunohistochemistry (IHC) and morphometry. In this investigation, TMAs were designed to include the main RCC subtypes (clear cell, collecting duct, papillary, chromophobe) and unclassified RCC. Histology grade using a grading system based on Fuhrman’s classification (**Table 1**) (16) was compared amongst RCC samples, and intensity of ICAD expression in the RCC subtypes was compared amongst RCC subtypes and with normal kidney. We were investigating whether profiling the protein expression of ICAD in TMAs could lead to a better understanding of RCC development.

**Table 1.** Fuhrman histological grade for RCC (16)

Fuhrman grade	Definition
1	Tumour cells with small (~10 µm), round, uniform nuclei without nucleoli
2	Tumour cells with larger nuclei (~15 µm); irregularities in nuclei and nucleoli when examined under high power (400)
3	Tumour cells with even larger nuclei (~20 µm); obvious irregular outline and prominent larger nucleoli at low power (100)
4	Tumour cells with bizarre, multilobed nuclei and heavy clumps of chromatin

## Methods

### Ethics approvals

Ethics approval was obtained from the University of Queensland Human Research Ethics Committee and the Princess Alexandra Hospital (PAH) Human Research Ethics Committee for these analyses (PAH HREC Approval 2006/189 and University of Queensland HREC Approval 2007000023). The original tissue blocks had been archived for research purposes with patient consent.

### General TMA protocol

Tumour biopsies were retrieved from selected regions of 121 archival RCC tumour tissue blocks of various subtypes (clear cell RCC, n = 95; papillary RCC, n = 11; chromophobe RCC, n = 6; collecting duct RCC, n = 4; and unclassified RCC, n = 5) from the PAH tumour tissue bank. The final number of biopsies used was 95. We acknowledge pathologist Dr J Perry-Keene for her work in re-classifying the archival RCC samples according to the 2004 WHO classification system (15). The paired normal kidney tissue was also used for each patient. The selected samples were precisely arrayed in new paraffin blocks to investigate and identify novel genes and genetic changes of potential importance in RCC. To carry out the TMA preparation, histology tissue blocks from the original pathology series were selected and a new histology section was cut so that there was a fresh haematoxylin and eosin (H&E)-stained section and a matching paraffin block (17). The sections were viewed under the microscope to select two representative areas of RCC or normal tissue from each sample block, at the Molecular and Clinical Pathology Research Laboratory, PAH. The areas of RCC were selected to best represent the RCC subtype classification. Fibrotic areas, glomeruli or large vessels were ignored. Normal tissue areas contained the proximal and distal tubular epithelium. Glomeruli and large vessels were not included where possible. Duplicate areas were selected from each section (and, therefore, paraffin block) to avoid any limitation from selection of a small core of tissue from a single area of one section. The TMA instrument used for the current project was a Galileo TMA CK3000 Tissue Microarrayer (Fantoli, Milan, Italy). The core punch needles used to remove the selected areas from the “parent” paraffin blocks were 0.6 mm in diameter and were from Beecher Instruments, Inc. (Sun Prairie, WI, USA).

### Immunohistochemistry

TMA sections were cut onto Superfrost Plus (ThermoFisher Scientific, Vic, Australia) histology slides for IHC. Primary antibody for ICAD was from AbCam Inc, Cambridge MA, USA (ab15202), and antibody dilution was 1:200. Positive tissue samples (human liver, kidney and gut arrays) were used to verify its activity in human tissue, and negative controls without primary antibody were prepared for each batch stain. Non-specific binding of peroxidase or antibody was blocked with 0.1% sodium azide in 0.3% hydrogen peroxide (H_2_O_2_) in Tris-buffered saline (TBS) (10 min), followed by 5% non-fat milk powder in TBS containing 0.05% Tween-20 (Blotto; 20 min), then a 1:100 dilution of normal swine serum in 1% bovine serum albumin (BSA) in TBS (5 min). Antibodies were diluted in 1% BSA in TBS. The IHC procedure was performed using a Bond-Max automated immunostainer (Vision BioSystems, Mount Waverley, Australia) in the Histology Unit, Queensland Institute for Medical Research, Brisbane, Australia, by Histology Manager Mr Clay Winterford. The kit used for IHC was a Bond Polymer Refine Detection kit (Vision BioSystems, Cat #DS9800). The chromogen was diaminobenzidine hydrochloride (DAB). Sections were lightly counterstained with haematoxylin and then dehydrated in a series of ethanol, cleared in xylene and mounted with glass coverslips in Depex (Sigma-Aldrich, MO, USA). Thus, the slides were stained in a batch in a constant environment, making comparisons in expression patterns amongst samples as controlled as possible.

### Morphometry

Digital images of the sections were captured using ScanScope from Aperio Digital Pathology systems (Leica Microsystems Pty Ltd, North Ryde, Australia). Up to three random fields of the same size were selected per RCC and paired normal kidney section, and jpg images were saved. A quantitative scoring system of overall expression, intensity and subcellular localisation of ICAD was performed. Image morphometry was analysed using ImagePro Plus image analysis software (version 4.1.29, Media Cybernetics, Silver Spring, MD), which automatically calculates the pixel area of DAB chromogen stained brown in each of the core images. In addition, a simple manual scoring system was used as a comparison with the automated morphometry. In this case, the analysis was carried out by viewing the actual sections using a light microscope and objective magnification of 40. Each core in each TMA was then scored manually for quantification of protein expression, based on the intensity of the staining and localisation of the expressed protein. Intensity and distribution were graded blinded to the subtype of RCC. For intensity, 1 represented no expression, and 2, 3 and 4 represented low, moderate and high expression, respectively, with a selection towards the lower of these scores if there was indecision on the score. For localisation, protein expression in membrane, cytoplasm or nucleus was recorded. Scores for RCCs were compared with scores with their paired normal tissue. Intensity of ICAD was also compared with grade of each RCC.

### Statistics

Various methods were used for statistics, depending on the types of analyses that were best for a particular set of comparisons. Associate Professor Zhiqiang Wang, Centre for Chronic Disease, University of Queensland, advised on some of the statistical analyses. Statistical methods employed were ANOVA with Bonferroni’s post hoc test, Student’s *t*-test, or Pearson χ^2^ with a Fisher’s exact p. A value of p < 0.05 was considered to be significant in each case.

## Results

### Expression of ICAD decreases in RCC and localises to the nucleus in clear cell RCC

**Figure 1.**
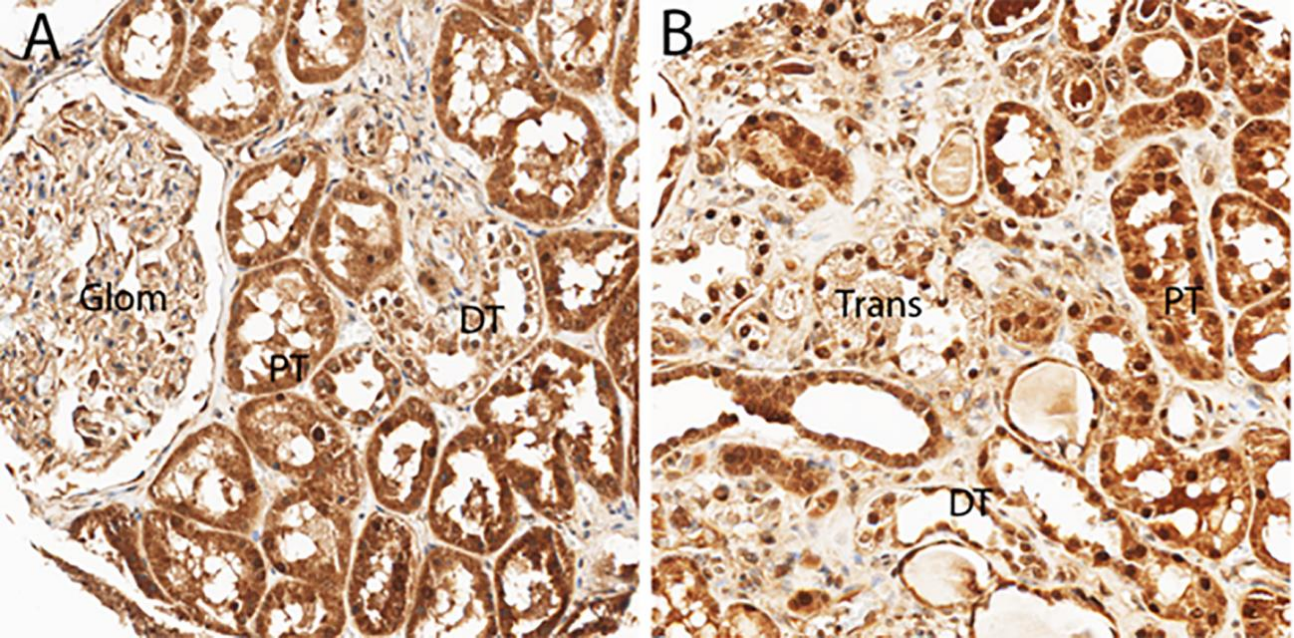
ICAD protein expression in normal kidney. In (**A**), expression was strong in the cytoplasm of the proximal tubular epithelium (PT) of normal kidney compared with distal tubular epithelium (DT) and glomerulus (glom). Nuclear expression is prominent in the distal tubule. In (**B**), there is a region that shows an apparent transition to clear cell RCC (trans), and within this region, nuclear localization of ICAD is intense, although cytoplasmic localization is still seen in the proximal tubular epithelium (PT).

ICAD is a downstream molecule of the caspases, usually inhibiting nuclear DNA fragmentation induced by CAD during apoptosis. ICAD is often located in cell nuclei and functions in the cytoplasm as an anchor for CAD or as a factor that enters the nucleus following caspase cleavage (6, 7, 14). In the current TMA analysis, **Figure 1A** demonstrates ICAD protein expression in normal kidney. Expression was strong in the cytoplasm of the proximal tubular epithelium of normal kidney, compared with distal tubular epithelium. The proximal tubular epithelium is said to be the origin of clear cell and papillary RCC, and distal tubular/collecting duct epithelium the origin of chromophobe and collecting duct RCC (3). Nuclear expression was prominent in the distal tubule. In **Figure 1B**, there is a region that shows an apparent transition to clear cell RCC (trans), and within this region, nuclear localisation of ICAD was intense, although cytoplasmic localisation was still seen in the proximal tubular epithelium.

**Figure 2.**
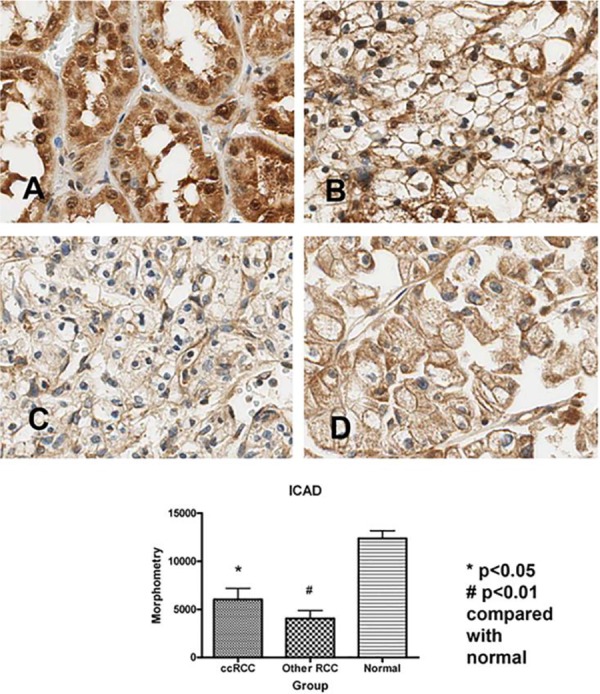
ICAD protein expression and morphometry in normal kidney versus RCCs. ICAD had very strong expression in the normal kidney (**A**), especially in the proximal tubular epithelium. Although clear cell RCC also had relatively strong expression (**B**), it was significantly reduced compared with normal tissue (p < 0.05). Likewise, other subtypes of RCC (**C** and **D** illustrate chromophobe and papillary, respectively) had significantly lower expression than that seen in the normal tissue (p < 0.01). There was some nuclear localisation in clear cell RCC (**B**), but in general no nuclear localisation was seen in other RCC subtypes (**C** and **D**). The morphometry results from the Image-Pro Plus software analyses for clear cell RCC versus all other non-clear cell RCC subtypes are plotted below the IHC. Expression (pixels) in all RCCs was significantly lower than that seen in the normal tissue. There was no significant difference in expression between clear cell RCC and all other RCC subtypes.

**Figure 2** compares ICAD expression between normal kidney and all RCC, using morphometry, and examples of IHC are given. Because of numbers (80% of samples were clear cell RCC versus only 20% for all other RCC subtypes), we analysed statistics by comparing expression in normal kidney with clear cell RCC and with all other non-clear cell RCCs grouped together. Although expression was significantly lower in all RCC subtypes, it was lowest in non-clear cell RCC. There was some nuclear localisation in clear cell RCC, but in general no nuclear localisation was seen in other RCC subtypes. The morphometry results from the Image-Pro Plus (Media Cybernetics, Inc, MD, USA) software analyses for clear cell RCC versus all other RCC subtypes are plotted below the IHC in **Figure 2**. ICAD had very strong expression in the normal kidney, especially in the proximal tubular epithelium. Using morphometry, expression (positive pixels) in all RCCs was significantly lower than that seen in the normal tissue.

**Figure 3.**
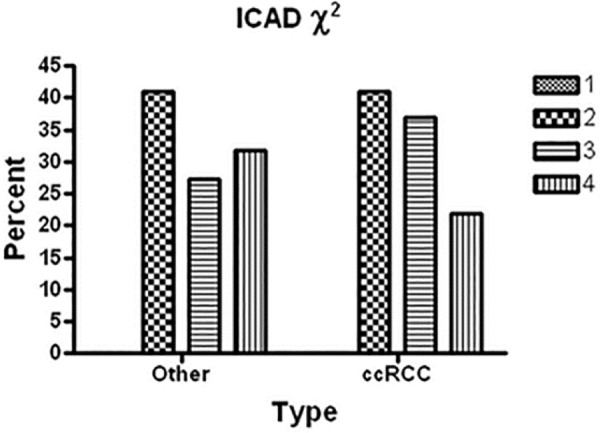
Graph of the χ^2^ results for RCC subtype with ICAD protein intensity. The ICAD χ^2^ analysis is shown, comparing percent of cases for clear cell RCC (ccRCC) with the other subtypes of RCC having differing intensity scores for protein expression (1 = no expression; 2–4 = low, moderate and high protein intensity). ccRCC was compared with all other RCC subtypes. The Pearson χ^2^ was 1.1437 with a p value of 0.564, and a Fisher’s exact p value was 0.553. Thus, there were no significant differences.

**Figure 4.**
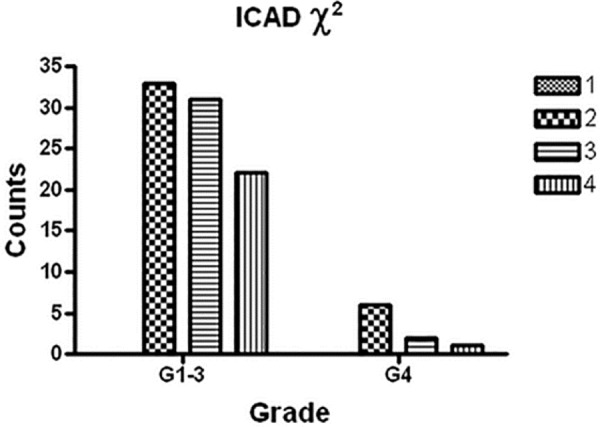
Graph of the 2 results for ICAD grade with protein intensity. The graphical summary of the numbers (Y axis = counts) of RCCs with a grade of 4 (G4) and those having grades 1 to 3 (G1–3) having a defined ICAD protein intensity (1 = no expression; 2–4 = low, moderate and high protein intensity). The Pearson 2 was 2.7417 with a p value of 0.254, and a Fisher’s exact p value was 0.327. Although there is clearly a decrease in ICAD intensity with high grade RCC, there was no significant difference.

**Table 2** and **Figure 3** demonstrate ICAD intensity scores compared between RCC subtypes using Pearson χ^2^. Clear cell RCC was compared with all other RCC subtypes The Pearson χ^2^ was 1.1437 with a p value of 0.564, and a Fisher’s exact p value was 0.553. Thus, there were no significant differences amongst RCC subtypes using this analysis. **Table 3** and **Figure 4** illustrate ICAD intensity scores when comparing Fuhrman’s histological grade 4 with grades 1–3. The Pearson χ^2^ was 2.7417 with a p value of 0.254, and a Fisher’s exact p value was 0.327. Again, there were no significant differences, although ICAD intensity was very low in grade 4 RCC.

**Table 2.** Comparison of RCC subtypes with ICAD protein intensity (Pearson χ^2^)

ICAD	Intensity score				
	1	2	3	4	Total
Rest of RCCs	0	9	6	7	22
	0.00	40.91	27.27	31.82	100.00
ccRCC only	0	30	27	16	73
	0.00	41.10	36.99	21.92	100.00
Total	0	39	33	23	95
	0.00	41.05	34.74	24.21	100.00

**Table 3.** Comparison of RCC tumour grade with ICAD protein intensity (Pearson χ^2^)

ICAD	Intensity score				
	1	2	3	4	Total
Grade 1–3	0	33	31	22	86
Grade 4 only	0	6	2	1	9
Total	0	39	33	23	95

## Discussion

While treating primary localised RCC with surgery is considered relatively successful, it is the metastases that are difficult to treat. The gene changes that develop in the primary cancers may determine the treatment resistance of metastases. Hanahan and Weinberg’s well-cited papers (4, 5) suggest that acquired capability of cancers to evade apoptosis is a key mechanism in progression of cancers. While evidence for this has developed principally from studies in mouse models and cultured cells, descriptive analysis of gene expression determining apoptosis in samples of human cancers is also a validated method for investigation. We used such a comparison in the current investigation.

Aberrations in the normal expression of genes, or the abnormal induction of genes not associated with normal renal homeostasis, could underlie RCC development and progression. In our previous research, RNA isolated from RCCs with low and high levels of apoptosis in cell culture experiments was subjected to microarray analysis (12). Genes differentially expressed between the high and low levels of apoptosis were grouped into apoptotic pathways and processes. Resistance to apoptosis can be acquired by cancer cells through a variety of strategies. Since the apoptotic machinery is made up of sensors responsible for monitoring the extracellular and intracellular environment for conditions of normality or abnormality, their altered expression may influence whether a cell lives or dies. ICAD is one of the final targets of the caspases, effector molecules of the apoptotic machinery (13, 18). Since ICAD acts to restrain CAD, its cleavage and inactivation by a caspase allows CAD to enter the nucleus and fragment the DNA, causing the characteristic DNA ladder in apoptosis. In our original microarray analysis of apoptosis in RCC, significant increases in RNA were found for ICAD (12), indicating its involvement in apoptosis resistance in the kidney cancer cells. Thus, TMA and IHC were implemented in the current study to investigate ICAD expression in human RCC samples compared with paired normal kidney.

In the TMA analysis, ICAD was significantly down-regulated in RCCs compared with normal kidney tissue where it was highly expressed mainly in the proximal tubule. ICAD normally inhibits DNA fragmentation and apoptosis (19); thus, the down-regulation in protein expression was unexpected in a cancer known for its resistance to apoptosis. However, similar down-regulation was also seen in proliferative colon cancer cells and was thought to be a mechanism for colon cancer cells to escape the late changes of apoptosis, including nuclear fragmentation and cell disintegration (20). These results in colon cancer may indicate a similar role in RCCs. The role of ICAD is indeed complex. As a downstream molecule of caspases, ICAD participates in nuclear DNA fragmentation during apoptosis. ICAD is typically located in cell nuclei (21), but it also functions in the cytoplasm as an anchor for CAD, or as a factor that enters the nucleus following caspase cleavage in order to activate resident endonucleases (22, 23). Isoforms of ICAD might function *in vivo* as tissue-specific modulators of an overall CAD activity available in the nuclei of cells, by controlling both the specific enzymatic activity and the amount of CAD in the nuclei (24). Some alterations in nuclear localisation of ICAD were seen in our study comparing normal kidney with RCC. These changes may also explain, at least in part, the unexpected down-regulation of ICAD in a cancer known to be resistant to apoptosis.

We believe it is now possible to include ICAD in a provisional apoptotic signalling circuitry in RCC. While information is incomplete, it is evident that most regulatory and effector components of apoptosis may be redundant in some cells. This redundancy holds important implications for the development of novel types of antitumour therapy, since tumour cells that have lost proapoptotic components are likely to retain ones that act in similar pathways. New technologies may be able to make the most of the information we have presented, to determine apoptotic pathways that are still operative in kidney cancer. From this information, new drugs may be developed that act on intact components of apoptotic signalling pathways in tumour cells, with substantial therapeutic benefit.

TMA technology should also be discussed. TMAs allow a molecule of interest to be studied within many small tissue pieces arrayed together into one paraffin block. The TMAs are prepared by relocating multiple tissue cores from conventional histologic paraffin blocks so that tissues from many patients can be analysed in the same slide. Thus, they are considered a highly efficient method for *in situ* screening of expression of proteins or mRNA (25–27). TMA technology clearly holds promise over other established methods for many different areas of research, especially in the field of cancer research (25). The disadvantage of this approach, however, is the lack a histomorphologic perspective in protein expression (28, 29) because tissue samples are small and may not be fully representative of a heterogeneic cancer such as RCC. In TMAs, IHC provides excellent localisation but lacks accurate quantification and normalisation to total cellular protein content (28, 30). However, as costs associated with assaying gene marker arrays increase, particularly for the testing of the efficacy of new drugs treatments, TMAs may well become a standard pre-clinical investigation tool (25, 31).

In summary, IHC of TMAs of paired human RCC and normal kidney tissue showed positive localized protein expression of ICAD, with detectable variation in expression levels of the proteins between normal and cancer tissues. Decreased ICAD expression in RCC compared with normal tissue was an unexpected finding; however, it is also known from other cancers that cells that lack ICAD, or express a caspase-resistant mutant of ICAD, may not have DNA fragmentation during apoptosis, or may die via other cell death pathways (14). The samples we used were also from patients who had received no therapy before resection of the cancer, and so we analysed endogenous ICAD expression. With a larger cohort for analysis, ICAD may prove useful as a marker of stage of progression of the cancer. However, it is not possible to hypothesise from these data whether treatment would have augmented expression above the levels of proteins described here. Further work is required to be completed with TMAs, using extended sampling of different subtypes and grades of RCC, to determine the prognostic value of ICAD.
